# The Soil Nutrient Environment Determines the Strategy by Which *Bacillus velezensis* HN03 Suppresses *Fusarium* wilt in Banana Plants

**DOI:** 10.3389/fpls.2020.599904

**Published:** 2020-11-16

**Authors:** Xiaoyan Wu, Ying Shan, Yi Li, Qinfen Li, Chunyuan Wu

**Affiliations:** ^1^Environment and Plant Protection Institute, Chinese Academy of Tropical Agricultural Sciences, Haikou, China; ^2^Key Laboratory of Integrated Pest Management on Tropical Crops, Ministry of Agriculture and Rural Affairs, Danzhou, China; ^3^Danzhou Scientific Observing and Experimental Station of Agro-Environment, Ministry of Agriculture and Rural Affairs, Danzhou, China; ^4^Hainan Engineering Research Center for Non-point Source and Heavy Metal Pollution Control, Haikou, China

**Keywords:** *Bacillus velezensis*, *Fusarium wilt*, soil nutrient environment, plant immunity, bacteria community

## Abstract

Biological control agents (BCAs) are considered as one of the most important strategies for controlling *Fusarium* wilt, and bioorganic fertilizer, in particular, has been extensively investigated. However, little is known regarding how a biocontrol microorganism affects the suppression mechanisms when combined with different amendments. In this study, a pot experiment was performed using banana plants to investigate the different mechanisms by which the biocontrol bacterium *Bacillus velezensis* HN03 (isolated from our laboratory) and amendments suppress *Fusarium* wilt. The incidence of banana wilt was decreased under HN03 and was reduced further when HN03 was combined with compost, particularly wormcast. In the suppression of *Fusarium* wilt, HN03 was found to influence the soil environment in various ways. HN03 increased the peroxidase level, which improves plant defense, and was highest when combined with wormcast, being 69 times higher than when combined with cow dung compost. The high accumulation of Mg and P in the “HN03 + wormcast” and Zn and Mn in the “HN03 + cow dung” treatments was negatively correlated with disease incidence. Furthermore, HN03 re-established the microbial community destroyed by the pathogen and further increased the level of suppression in the wormcast. HN03 also enhanced the functional traits of the soil, including defensive mechanism-related traits, and these traits were further enhanced by the combination of HN03 + wormcast.

## Introduction

Soil-borne pathogens are the causal agents of several plant diseases of global importance and cause substantial economic losses (Ruiz-Romero et al., 2018). *Fusarium* wilt disease has become a serious threat to Cavendish banana (*Musa acuminata* L. AAA group, cv. Cavendish), which is the most widely planted cash crop in South China, because this cultivar is susceptible to the soil-borne pathogen *Fusarium oxysporum* f. sp. *cubense* tropical race 4 (*FOC*4) (Shen et al., 2015).

The biological control of *Fusarium* wilt by antagonistic bacteria offers a promising strategy and has attracted major research attention (Fu et al., 2017; Xiong et al., 2017; Sun et al., 2018). Biological control agents (BCAs) reduce infections or disease through antibiosis, parasitism, or competition (for space and/or nutrients), induction of plant local/systemic resistance, plant growth promotion, or changes in soil/plant microbiota (Bubici et al., 2019). Several antagonistic bacteria, such as *Bacillus*, *Trichoderma*, *Pseudomonas*, non-pathogenic *Fusarium*, and *Penicillium* strains, have been evaluated as possible means of controlling *Fusarium* wilt of banana (Raza et al., 2017); however, no single biological product can be recommended for widespread use to control this disease (Dita et al., 2018). It is thought that compost application can help reduce pathogen attack and improve soil health and nutrient levels (Mehta et al., 2014), but the application of compost alone often results in inconsistent levels of disease control (Lang et al., 2011). However, the manipulation of compost by inoculation or enrichment with specific antagonists to produce bio-organic fertilizer is believed to be a more efficient means of controlling soil-borne disease than the use of a single antagonistic microbe or compost type (Shen et al., 2013).

Recently, some studies have evaluated combinations of antagonistic microbes and compost in controlling *Fusarium* wilt (Fu et al., 2016, 2017; Huang et al., 2019). Changes to the soil microbial community are considered as the main mechanism through which bio-organic fertilizer promotes soil suppression of disease (Shen et al., 2013; Huang et al., 2017). The reported mechanisms include inhibiting soil-borne pathogen growth, reducing the population of pathogens, recovering the microbial populations damaged by pathogens, and altering the composition of the bacterial community (Lang et al., 2011; Qiu et al., 2012; Zhao et al., 2014). Previous studies have also showed that the manipulation of soil P level by organic fertilizer is one possible mechanism by which bio-organic fertilizer reduces the incidence of *Fusarium* disease (Yergeau et al., 2006). Some other studies demonstrated that bio-organic fertilizer could induce plant suppression of pathogens by activating the defense enzymes of the plant (Wang et al., 2015), such as the enhancement of peroxidase (POD) activity when controlling *Fusarium* wilt of pepper following bio-organic fertilizer application (Wu et al., 2015). Though previous studies suggest similar mechanisms of action for antagonistic bacteria, compost, and bioorganic fertilizer, such as the induction of plant resistance and regulation of the microbial community (Dita et al., 2018), few studies have focused on how these factors interact to suppress pathogens, and, particularly, how BCAs alter the functional and nutritional characteristics of microbial communities to enhance disease control.

Understanding the action modes of BCAs is essential for exploiting their potential for effective disease management (Bubici et al., 2019). Previous studies showed that improvements in seed growth, nutrient uptake, and soil microflora may be related to the strain (*Bacillus* spp.) inoculated into the compost (El-Hassan and Gowen, 2006). Additionally, BCAs combined with compost might demonstrate better biocontrol due to additive, or even synergistic, interactions between BCA and compost. For example, the combination of compatible supplementary sources with a biocontrol strain can improve phytopathogen suppression to a reliable level (Regassa et al., 2018). However, our understanding of the role of BCAs in mediating the control mechanisms of banana *Fusarium* wilt under different nutrient environments in the soil remains limited. Here, we focus on the specific interactions between a BCA (*Bacillus velezensis* HN03) and its soil nutrient environment, aiming to construct a more comprehensive understanding of the role of HN03 in mediating nutrients and structuring the functional characteristics of microbial communities, as well as its role in inducing defense enzymes to control banana *Fusarium* wilt in plants and soil.

The specific interactions between BCA and the highly complex soil environment are difficult to discern when investigating a soil-borne disease, as their understanding requires an assessment of the broader influences on plant and soil suppression. Recently, Illumina sequencing technology has been widely used in microbial communities associated with banana *Fusarium* wilt. Unique distributions of bacteria and fungi were observed in diseased and disease-free soil samples from banana fields (Zhou et al., 2019). The importance of microorganisms in soil nutrient cycling and their role in plant nutrition is well established (Kucey et al., 1989), and some nutrients are reported to be related to plant disease defense (Siddiqui et al., 2015). If BCA can mediate the nutrient profile to defend against banana *Fusarium* wilt in different environments, then different nutrients would be found to accumulate in the soil and plants. Moreover, if HN03 can mediate the structural functional characteristics of microbial communities to defend against banana *Fusarium* wilt under different environments, then different suppression levels and functions would be found in the soil. Finally, BCA mobilizes different composts to construct a new suppression system in the soil and plant, and in this new system, the defense abilities of HN03 or compost are strengthened. Furthermore, new defense mechanisms are formed in this complex system.

Here, we evaluated the comprehensive mechanism of suppression of *Fusarium* wilt by BCA in different environments. We hypothesized that the suppression mechanisms are involved in plant immunity, soil microbial community, trophic interactions, and specific functional traits. In order to test our hypothesis, a new biocontrol bacterium, *Bacillus velezensis* HN03, with strong adaptability to the environment and wide application to various plant soil-borne diseases, was isolated and identified. Furthermore, pot experiments with banana plants were designed to determine the capacity of HN03 to suppress *FOC*4 among different nutrient environments, following which HN03-mediated transformation of the main mechanism involved in improving suppression in compost was explored. Collectively our data showed that HN03 regulated the changes in the main suppression mechanisms to control banana *Fusarium* wilt in different environments, thus highlighting the significance of BCA and appropriate carriers in controlling soil-borne disease.

## Materials and Methods

### Bacterial Strain

The bacterial strain designated HN03 used in this study was isolated from laterite soil in our laboratory, which was collected from rhizospheric soil of healthy bananas in Haikou City, Hainan Province, China, located at 19°56′34″N, 110°04′27.25″E. The isolation of HN03 using a method from Li et al. (2017).

### Testing for Antifungal Activity Against Fungal Pathogens

Twelve strains of fungal pathogens (Supplementary Table 1) were selected from the Key Laboratory of Integrated Pest Management on Tropical Crops, including 10 strains of soil-borne pathogens and 2 strains of common pathogens. The antagonism of HN03 toward the pathogens was assessed by measuring the inhibition of the growth rate using a method from Rajaofera et al. (2017) with a slight modification. A 6 mm mycelial disk of a pathogenic fungus collected from the edge of an actively growing colony was placed into the center of a PDA (potato dextrose agar) plate. An inoculum of HN03 bacterial cells (0.2 μL) was delivered around the periphery of the target fungus within a radius of 2.5 cm.

### Identification of the Bacterial Strain

The HN03 strain was physiologically and biochemically characterized using a 96-well plate test system (Kämpfer et al., 1991) by a GenIII Microplate (Biolog, Hayward, United States). Characterizations including carbon source utilization and antibiotic resistance are listed in Supplementary Table 2, and other characteristics are listed in Supplementary Table 3 and were tested according to the instructions of Bergey’s Manual of Systematic Bacteriology (Sneath, 1986; Wayne, 1986).

Molecular biological tests were performed to verify the identification based on the physiological and biochemical tests. To determine the phylogenetic affiliation of strain HN03 for molecular identification, genomic DNA was extracted using a Bacterial Genomic DNA Extraction Kit (Solarbio, Beijing, China) and purified using a Universal DNA Purification Kit (Tiangen, Beijing, China). The 16S ribosomal gene sequence of strain HN03 was amplified using primers 27F and M1492R as described by Ma et al. (2016). The PCR product was cloned into a pEasy-T1 cloning vector (TransGen, Beijing, China) for sequencing. The sequence of strain HN03 was submitted to GenBank to search for similar sequences using the BLAST algorithm^1^ and was compared with sequences available on the EzTaxon-e server provided by EzTaxon^2^ (Kim et al., 2012).

DNA–DNA hybridization was also performed with the strain with the closest similarities in biochemical and physiological characteristics and 16S rRNA gene sequences. The levels of DNA–DNA hybridization were determined using a modified optical renaturation method described by De et al. (1970) and Gillis et al. (1970).

### Bio-Organic Fertilizer Preparation

A single colony of the HN03 strain was grown in 50 mL of nutrient broth at 30°C for 24 h (at 200 rpm), which was then inoculated in nutrient agar at a 1:100 (v/v) ratio and grown at 30°C for 2–3 d (at 200 rpm) before harvesting. Bacterial cells were collected by centrifugation at 8,000 (× g) for 10 min and were resuspended with the same volume of distilled water at a final concentration of 10^8^ CFU/mL, which was used as the bacterial cell inoculum for antifungal activity tests and bio-organic fertilizer preparation.

A total of 600 earthworms, *Eisenia fetida*, were grown in a box with a 4,500 g mixture of cattle manure and sawdust for approximately 1.5 months. Then the earthworms were removed, and the processed mixture was air dried, crushed, and passed through a 2 mm mesh sieve to obtain the wormcast used in the current study. The cow dung was fermented and composted for about 1 month in a box while covered by a plastic film. The dung was air dried, crushed, and passed through a 2 mm mesh sieve.

### Pot Experiment

Pot experiments were performed from July to October 2017 in the greenhouse of the Chinese Academy of Tropical Agricultural Sciences, located in Hainan, China. Banana seedlings (*Musa acuminata* L. AAA group, cv. Cavendish) with 3–4 true leaves, weighing 3.30 ± 0.16 g and 8.29 ± 0.31 g in mass in the above- and belowground parts, respectively, and approximately 5.16 ± 0.12 cm in height, were used for the experiment.

*FOC*4 was grown in PDA liquid culture at 28°C for 4–5 d (at 200 rpm). The culture was filtered through a sterile pledget to obtain a spore suspension, which was then diluted to a concentration greater than 5 × 10^5^ spores/mL with distilled water.

The soil used in the pot experiment, classified as laterite (clay), was collected from Haikou City, Hainan Province, China, located at 19°56′38.3″N, 110°28′42.9″E, and had the following properties: pH 7.1; organic matter (OM) 7.4 g kg^–1^; available nitrogen (AN) 18.9 mg kg^–1^; available phosphorus (AP) 3.58 mg kg^–1^; available potassium (AK) 78.9 mg kg^–1^; total nitrogen (TN) 701.99 mg kg^–1^; total phosphorus (TP) 278.38 mg kg^–1^; and total potassium (TK) 4601.47 mg kg^–1^. The properties were measured according to section “Resistance Activity and Mineral Nutrient Assays for Leaf and Soil.”

Banana seedlings were grown in plastic pots (7 cm diameter, 16-m depth) with 2,000 g of culture medium. All plant roots were treated with the *FOC*4 spore suspension for 20 min after trimming the roots to 10 cm, except the healthy controls, which were treated with water for 20 min after root trimming. To explore the action modes of HN03 and its nutrient environments to soil-borne disease, a factorial design (2 × 3 + control) was set to two levels of inoculation (with HN03 and without it) and three levels of amendments (without amendment, wormcast and cow dung compost), plus the control S. The pot experiment included the following seven treatments: (1) healthy control (S): healthy plants were grown in untreated soil that was irrigated with 500 mL of water every 7 d; (2) disease control (S + F): *FOC*4-infected plants were grown in soil that was irrigated with 500 mL of water every 7 d; (3) HN03 treatment (S + F + B): *FOC*4-infected plants were grown in soil that was irrigated with 500 mL of 50-fold diluted HN03 bacterial cell inoculum every 7 days; (4) HN03 amended with wormcast treatment (S + F + B + EW): *FOC*4-infected plants were grown in soil containing 10% (w/w) of the wormcast bio-organic fertilizer that was irrigated with 500 mL of 50-fold diluted HN03 bacterial cell inoculum every 7 days; (5) wormcast treatment (S + F + EW): *FOC*4-infected plants were grown in soil containing 10% (w/w) of the wormcast bio-organic fertilizer that was irrigated with 500 mL of water every 7 days; (6) HN03 amended with cow dung compost treatment (S + F + B + CD): *FOC*4-infected plants were grown in soil containing 10% (w/w) of the cow dung compost bio-organic fertilizer that was irrigated with 500 mL of 50-fold diluted HN03 bacterial cell inoculum every 7 days; and (7) cow dung compost treatment (S + F + CD): *FOC*4-infected plants were grown in soil with 10% (w/w) of the cow dung compost bio-organic fertilizer that was irrigated with 500 mL of water every 7 days. Therefore, every 7 days, plants in treatments S, S + F, S + F + EW, and S + F + CD were irrigated with water, whereas plants in treatments S + F + B, S + F + B + EW, and S + F + B + CD were irrigated with the same amount of diluted HN03 bacterial cell inoculum. The seedlings were grown in a greenhouse without any pesticides or fertilizers for 90 d; the temperature ranged from 22 to 30°C, and relative humidity was from 75 to 85%. One plant was planted per pot, with three pots per replicate and three replicates per treatment, resulting in a total of 63 seedlings for the seven treatments.

### Disease Incidence and Plant Growth Assessment

Seedling infection by *FOC*4 was recorded daily, and disease development investigated as disease incidence (DI) was recorded on a 5-grade scale from 0 to 4 as described by Huang et al. (2014): 0 = no wilting, 1 = 1–25% wilting, 2 = 26-50% wilting, 3 = 51-75% wilting, and 4 = 76-100% wilting or dead. The DI value of the different treatments was calculated according to the method described by Huang et al. (2014). The biocontrol efficacies (BE) were calculated as described by Tan et al. (2015).

Seedling pseudo-stem height (distance from the base of the plant to the point of the youngest emergent leaf) was measured. The fresh plants were dried in an oven at 70°C for 72 h until constant weight was reached, and the dry weight (weight of aboveground parts including the leaves and banana cauloid and weight of belowground parts including the banana corm and root) was measured on a scale (± 0.01 g).

### Resistance Activity and Mineral Nutrient Assays for Leaf and Soil

After 90 d, leaf samples and soil samples were obtained from three biological replicate pots. The first and third leaves from the apex of the dominant stem were combined and used for plant tissue analysis.

The POD content in the banana seedlings was analyzed using their respective assay kits (Solarbio, Beijing, China).

The leaves detached from the stems were cleaned dried and ground using a ball mill. Tissue nitrogen (N) content was measured in a Carlo Erba NA 1,500 C/N analyzer (Milan, Italy) (Steiner et al., 2008). Tissue phosphorus (P) content was determined using Mo–Sb colorimetry (Kowalenko and Babuin, 2007). For mineral nutrients, potassium (K), calcium (Ca), magnesium (Mg), manganese (Mn), iron (Fe), and zinc (Zn) content were determined by an atomic absorption spectrophotometer (PerkinElmer PinAAcle 900T, Waltham, MA, United States) after extraction (Muhammad et al., 2010).

Soil samples were collected using a horticultural shovel and were air dried and crushed to pass through a 2-mm mesh sieve after removing the plant parts. Loss-on-ignition as a rough measure of soil OM was determined by igniting 2 g of soil in a muffle furnace at 600°C for 6 h followed by overnight cooling (Salehi et al., 2011). TP, AP, TN, AN, TK, and AK were measured according to Roiloa et al. (2015). Exchangeable Mg and Ca were determined by an atomic absorption spectrophotometer (PerkinElmer PinAAcle 900T, Waltham, MA United States) after extraction (Olorunfemi et al., 2018).

### Soil DNA Extraction, PCR, and Sequencing

Total soil DNA was extracted from samples using a Power Soil DNA Isolation Kit (MO BIO Laboratories, Carlsbad, CA, United States) according to the manufacturer’s protocol. DNA quality and quantity were assessed based on the ratios of 260/280 and 260/230 nm. The DNA was stored at −80°C until further processing. The V3–V4 region of the bacterial 16S rRNA gene was amplified (forward primer, 5′-ACTCCTACGGGAGGCAGCA-3′; reverse primer, 5′-GGACTACHVGGGTWTCTAAT-3′) (Mori et al., 2014) and combined with adapter sequences and bar code sequences. The ITS1 region of the fungi was amplified (forward primer, 5′-CTTGGTCATTTAGAGGAAGTAA-3′; reverse primer, 5′-GCTGCGTTCTTCATCGATGC-3′) (Fouquier et al., 2016) and combined with adapter sequences and bar code sequences. The PCR amplification was conducted in 50-μL reactions containing 10 μL of 5 × PCR buffer, 0.2 μL of Q5 High-Fidelity DNA Polymerase (NEB, Ipswich, United States), 10 μL of High GC Enhancer, 1 μL of dNTP, 10 μM each primer, and 60 ng of genomic DNA. The PCR amplification conditions were set as follows: an initial denaturation at 95°C for 5 min; followed by 15 cycles consisting of 95°, 50°C, and 72°C for 1 min; and a final extension step at 72°C for 7 min. The PCR products from the first round of PCR were purified through VAHTSTM DNA Clean Beads (Vazyme, Nanjing, China) and were used as the template in the second round of PCR. A second round of PCR was then performed in a 40-μL reaction containing 10 μL of PCR products from the first round of PCR, 20 μL of 2 × Phusion HF MM, 8 μL of ddH_2_O, and 10 μM each primer. The PCR amplification conditions were set as follows: an initial denaturation at 98°C for 30 s; followed by 10 cycles consisting of 98°C for 10 s, 65°C for 30 s, and 72°C for 30 s; and a final extension step at 72°C for 5 min. Finally, all PCR products quantified by the Quant-iT^TM^ dsDNA HS Reagent (Invitrogen, Carlsbad, CA, United States) were pooled and used for high-throughput sequencing analysis of bacterial rRNA genes in an Illumina HiSeq 2,500 platform (Illumina, Santiago, United States) (2 × 250 paired ends) at Biomarker Technologies Corporation, Beijing, China.

### Statistical Analyses

All data were analyzed using the statistical program IBM SPSS version 19 (2010 SPSS, Inc., Chicago, IL, United States). Data are presented as the mean ± SD. Non-normally distributed variables were normalized using Bloom’s Formula (Bo et al., 2009), and means were compared using Tukey’s test at 5%. Differences were analyzed using one-way ANOVA and least significant difference (LSD) tests. Differences at *P* < 0.05 were considered statistically significant. PCA and RDA were conducted using Canoco version 5 (2012, Biometry, Plant Research International, the Netherlands). Sequence analysis was performed by the UPARSE version 10.0 software (2013, CA, United States) package using the UPARSE-OTU and UPARSE-OTUref algorithm (Edgar, 2013). In-house Perl scripts were used to analyze alpha (within samples) and beta (among samples) diversity. Sequences with ≥ 97% similarity were assigned to the same OTUs, and heatmaps based on the retained OTUs were constructed based on the Bray–Curtis distance and Binary–Jaccard distance (Winstanley et al., 2005) using BMKCloud^3^. In order to compute alpha diversity, we rarified the OTU table and calculated five metrics: Chao1 and ACE estimates for species abundance; Observed species estimates for the number of unique OTUs found in each sample; and Simpson and Shannon indexes using Mothur v1.30 (2013, MI, US) (Schloss et al., 2009). The smaller the Simpson index, the higher the community diversity. A greater Shannon index indicates a higher community diversity. Rarefaction curves were generated based on these three metrics (Wang et al., 2012; Zhao et al., 2016). Microorganism features distinguishing specific microbiota were identified using the LDA LEfSe method^4^ for biomarker discovery, and an alpha significance level of 0.05 and an effect-size threshold of 2 were used for all biomarkers (Jiang et al., 2015). To determine the statistical differences of bacterial functions between the treatments, Statistical Analysis of Metagenomics Profile v2.1.3 (STAMP) software based on the Clusters of Orthologous Groups (COG) was used (Parks et al., 2014). Spearman’s correlation analysis was performed to determine the links between the bacterial community and the environmental factors (AK, AP, AN, TK, TP, TN, OM, Ca, and Mg) using IBM SPSS version 19 (2010 SPSS, Inc., Chicago, IL, United States). The significance tests of Monte Carlo permutations were conducted to construct the appropriate models of the bacteria–environment relationships using Canoco version 5 (2012, Biometry, Plant Research International, Netherlands).

## Results

### Identification of the Bacterial Strain

Strain HN03 was phenotypically characterized as a Gram-positive, rod-shaped, endospore-forming bacterium with positive oxidase activity. The strain produced proteases, cellulase, and amylase and was positive for the Voges–Proskauer test (Supplementary Table 1). HN03 grew well in the temperature range of 15–50°C, a NaCl content of 0–10% (w/v), and on medium amended with glucose, sucrose, glycerol, and D-mannitol. HN03 could assimilate carbon from a wide variety of sources and demonstrated good growth with various nitrogen source extracts (Supplementary Table 2).

**FIGURE 1:**
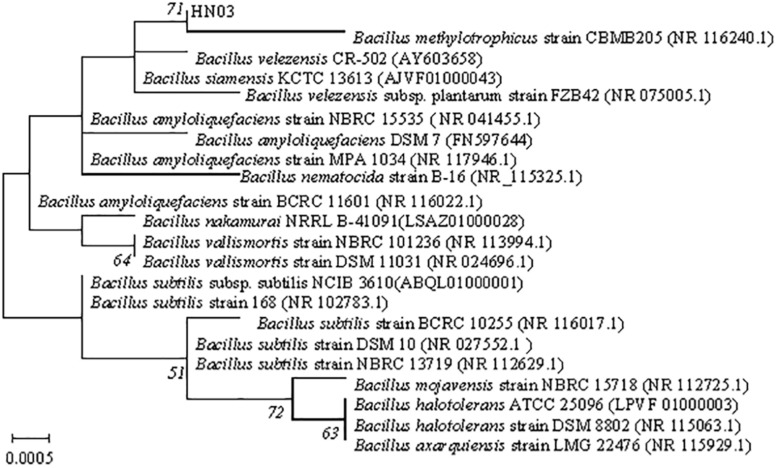
Maximum-likelihood phylogenetic tree based on partial 16S rRNA gene sequences (1,424 bp) showing the position of strain HN03. Bootstrap values (>50%) based on 1,000 replications are shown at branch nodes. Bar = 0.0005 substitutions per nucleotide position.

BLAST analysis revealed a high level of similarity (99%) to the sequence of *B. velezensis*, as well as to those of other *Bacillus* species. Pairwise sequence similarities of the 16S rRNA genes of HN03 to the most closely related type strains revealed a high sequence similarity of 99.93% in both strains with that of *B. velezensis* and *B. siamensis*. The sequences of 16S rRNA of the HN03 strain were deposited in GenBank under accession No. MF155192. In the maximum-likelihood tree (Figure 1), HN03 was within a group containing *B. methylotrophicus* strain CBMB205, adjacent to *B. velezensis* CR-502, *B. siamensis* KCTC 13613, and *B. velezensis* subsp. *plantarum* strain FZB42, and was similar to the strain *Bacillus amyloliquefaciens* DSM 7. The biochemical and physiological data of those strains were reported in previous studies (Ruiz-Garcia et al., 2005; Madhaiyan et al., 2010; Sumpavapol et al., 2010; Borriss et al., 2011) and are compared with HN03 in Supplementary Table 2. The data showed that *B. velezensis* subsp. *plantarum* strain FZB42 had the highest similarity with HN03.

DNA–DNA hybridization showed that HN03 had 90.9% DNA–DNA relatedness to the closest reference isolate *B. velezensis* FZB42^*T*^. According to these results, HN03 is closely related taxonomically to the plant-associated strains of *B. velezensis.*

### Antifungal Activity of HN03

**FIGURE 2:**
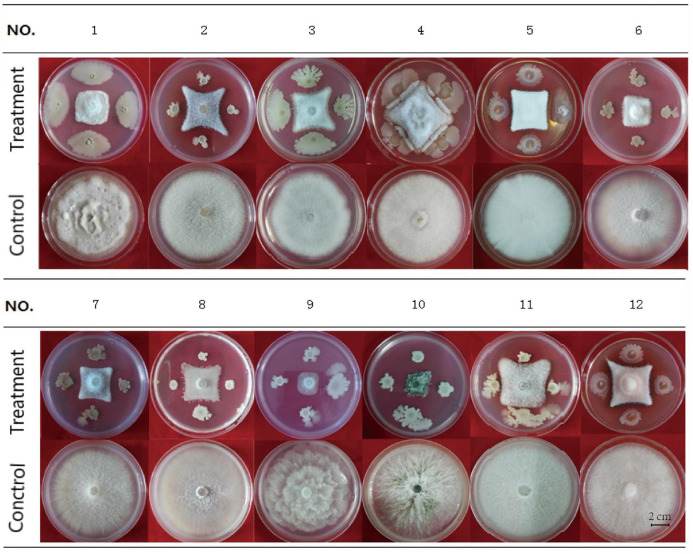
Antifungal activity of HN03 against 12 pathogenic strains of fungi. A dual culture assay was used to determine the *in vitro* inhibition of mycelial growth. The fungal pathogens were co-cultured with the bacterial strain HN03 on potato dextrose agar (treatment, pathogen + HN03; control, pathogen only). No. 1, *FOC*4 (Hainan); No. 2, *FOC*4 (Vietnam); No. 3, *Fusarium oxysporum* f. sp. cubense 1 (Hainan); No. 4, *Fusarium solani* of Noni; No. 5, *Fusarium solani* of Annona squamosal; No. 6, *Fusarium oxysporum* f. sp. *radicis lycopersic*; No. 7, *Fusarium oxysporum* f. sp. *melonis*; No. 8, *Fusarium oxysporum* f. sp. *niveum*; No. 9, *Phytophthora nicotianae*; No. 10, *Colletotrichum gloeosporioides*; No. 11, *Fusarium solani* of Medicago; No. 12, *Fusarium solani* of Annona squamosal (Hainan).

HN03 had inhibitory activity against a broad spectrum of fungal pathogens and suppressed the mycelial growth of the 12 tested strains (Figure 2), 10 of which were soil-borne pathogens and 2 of which were common pathogens. The activity of HN03 against *FOC4* (Vietnam) was 68.79%, followed by *FOC*4 at 68.47% (Supplementary Table 3).

### Efficiency Against Banana *Fusarium* wilt of HN03 and Compost

**FIGURE 3:**
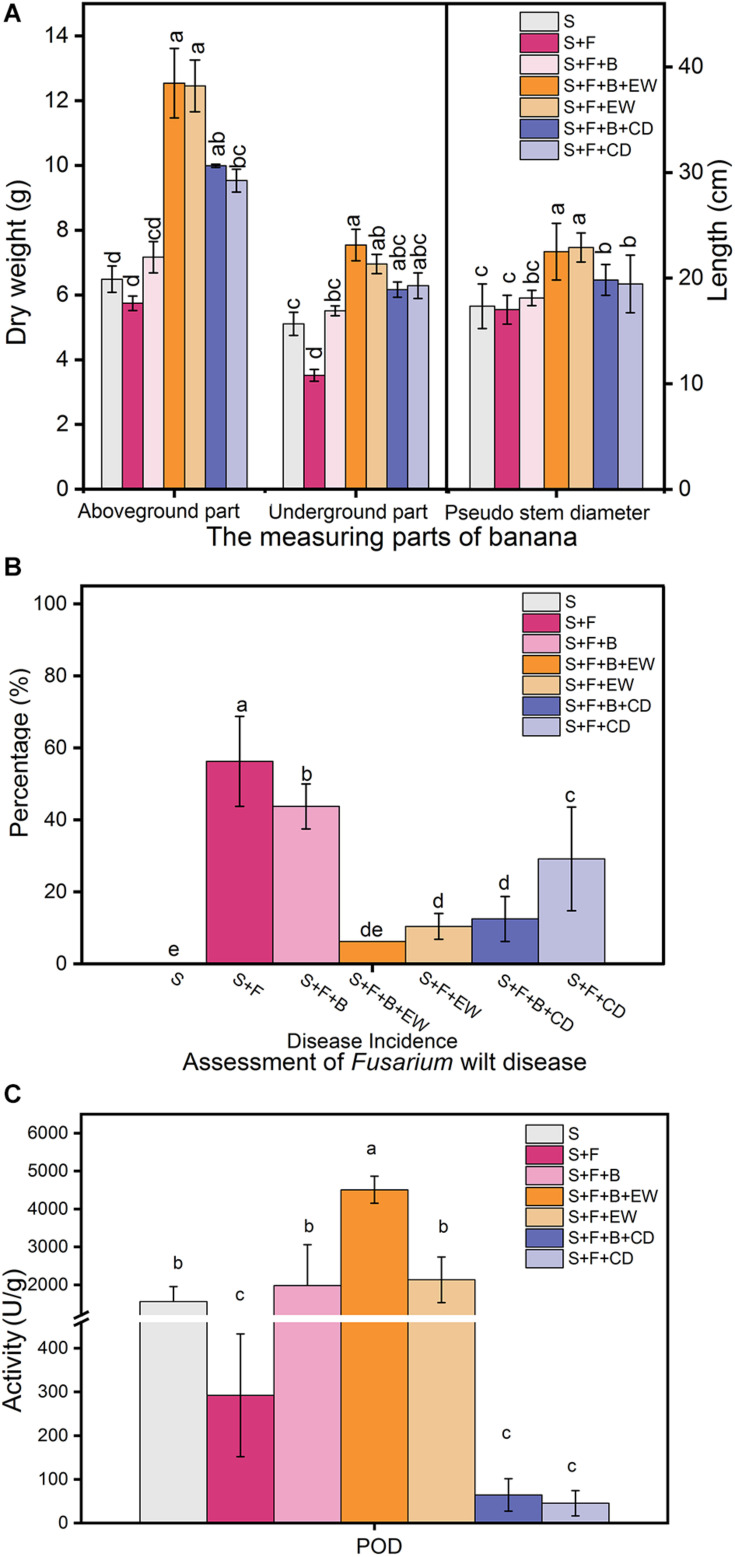
Effects of the treatments on **(A)** dry weights of the aboveground and belowground parts and the height (cm) of the pseudo-stem of banana, **(B)** assessment of *Fusarium* wilt disease, and **(C)** contents of peroxidase (POD) in banana leaves in the pot experiment. Values are the mean ± SD of three replicates. S, healthy control (healthy soil); S + F, disease control (healthy soil + *FOC*4); S + F + B, healthy soil + *FOC*4 + HN03 treatment; S + F + B + EW, healthy soil + *FOC*4 + HN03 + wormcast treatment; S + F + EW, healthy soil + *FOC*4 + wormcast treatment; S + F + B + CD, healthy soil + *FOC*4 + HN03 + cow dung compost treatment; S + F + CD, healthy soil + *FOC*4 + cow dung compost treatment. Bars sharing the same letter are not significantly different based on Duncan’s multiple range test at *P* < 0.05.

Irrigation with HN03 rendered the dry weight of the underground parts significantly greater than that of the plants in the disease control (S + F) treatment and was highest in treatment “S + F + B + EW” (Figure 3A). The wormcast was a much better supplement than cow dung when measured based on the weight of the aboveground parts and the pseudo-stem height of the banana plants, while HN03 application weakened the significant differences observed in the aboveground parts between cow dung and wormcast.

In comparison to the disease control treatment (S + F), both the HN03 treatments (S + F + B) and compost treatments (S + F + EW and S + F + CD) significantly reduced the disease incidence (DI) of banana *Fusarium* wilt (Figure 3B). The “S + F + B + EW” treatment showed the lowest DI among the *FOC*4-treated treatments, with a value of 6.25%. Interestingly, there was a significant difference in DI between the “S + F + CD” and “S + F + B + CD” treatments, but no significant difference in DI between “S + F + EW” and “S + F + B + EW.”

### Peroxidase in the Leaves

As shown in Figure 3C, the addition of HN03 increased the POD content in the banana seedlings, and the POD content of the plants in treatment “S + F + B” was approximately seven times higher than that in treatment “S + F.” When HN03 was combined with wormcast in treatment “S + F + B + EW,” the POD content of the plants increased significantly and was the highest among the seven treatments, and was more than two times that in the plants in treatment “S + F + EW.” The wormcast was a much better supplement than cow dung, and the POD content of the plants in treatment “S + F + B + EW” was 69 times higher than that in treatment “S + F + B + CD”.

### Mineral Nutrients in the Leaf and Soil

**FIGURE 4:**
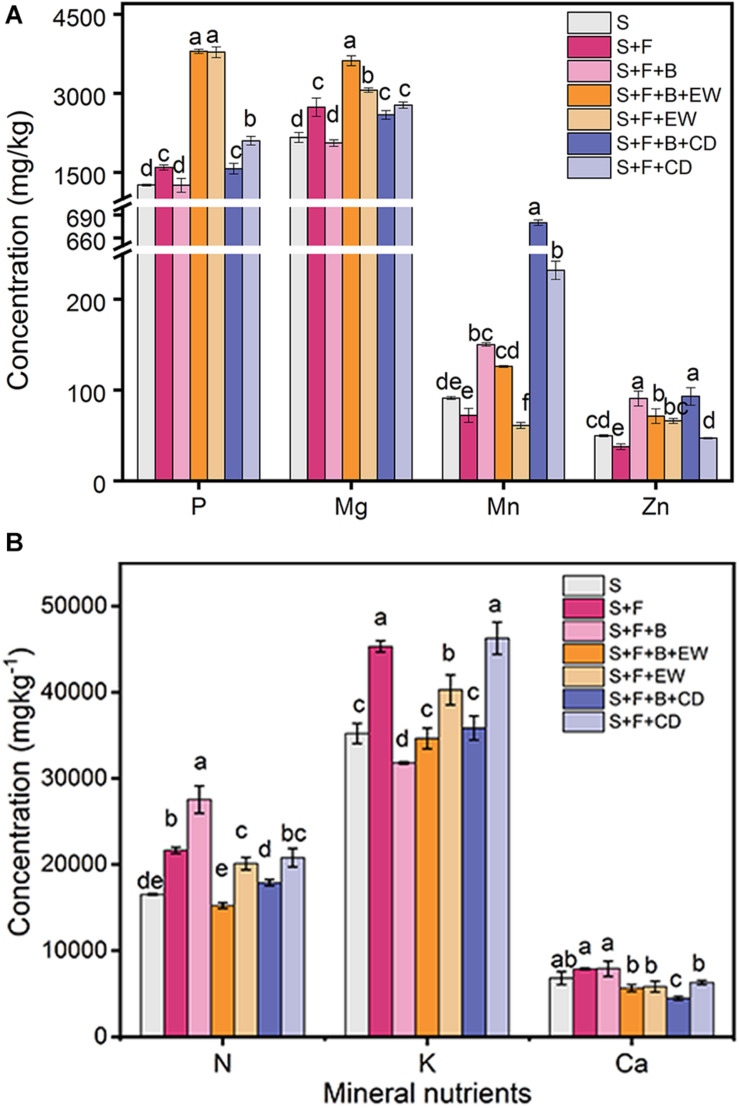
Contents (mg/kg) of mineral nutrients in banana leaves in the pot experiment. **(A)** P, Mg, Mn, and Zn; **(B)** N, K, and Ca. Values are the mean ± SD of three replicates. S, healthy control; S, healthy control (healthy soil); S + F, disease control (healthy soil + *FOC*4); S + F + B, healthy soil + *FOC*4 + HN03 treatment; S + F + B + EW, healthy soil + *FOC*4 + HN03 + wormcast treatment; S + F + EW, healthy soil + *FOC*4 + wormcast treatment; S + F + B + CD, healthy soil + *FOC*4 + HN03 + cow dung compost treatment; S + F + CD, healthy soil + *FOC*4 + cow dung compost treatment. Bars sharing the same letter are not significantly different based on Tukey’s test at *P* < 0.05.

The addition of HN03 increased the content of Mn in the banana leaves in treatments “S + F + B,” “S + F + B + EW,” and “S + F + B + CD,” which had significantly higher contents than their comparable treatments without HN03 (S + F, S + F + EW, S + F + CD), and the highest contents were found in “S + F + B + CD.” The content of Zn was high in the treatment “S + F” and highest in “S + F + B + CD.” The contents of P and Mg in the banana leaves in the wormcast treatments (“S + F + B + EW” and “”S + F + EW”) were significantly higher than those in the cow dung treatments (“S + F + B + CD” and “S + F + CD”), and the content of Mg in “S + F + B + EW” was significantly higher than in the other treatments (Figure 4A). In addition, the contents of N, K, and Ca of the leaves in all treatments were not significantly different from each other (Figure 4B).

**FIGURE 5:**
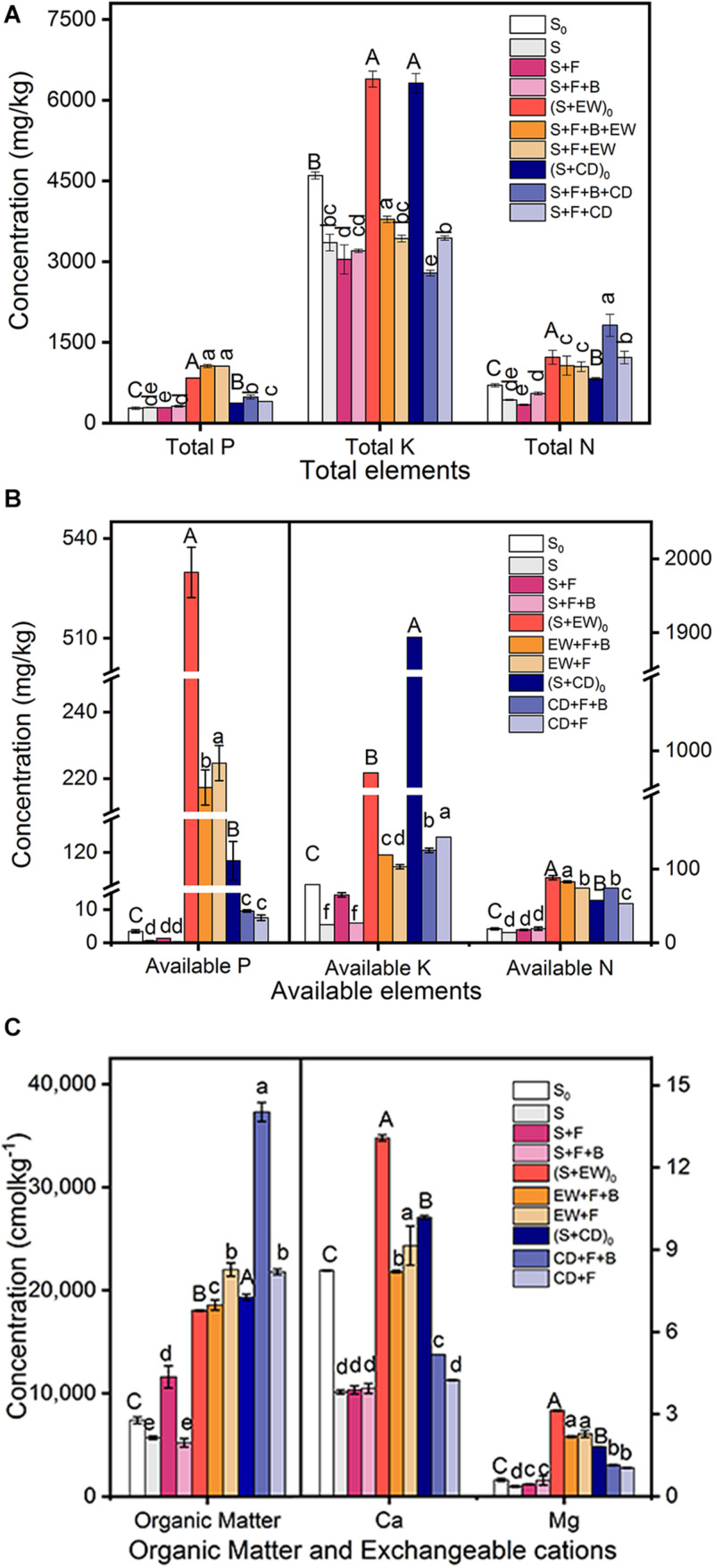
Contents (mg/kg) of mineral nutrients in the soil in the pot experiment. **(A)** Total elements: TP, TK, and TN; **(B)** available elements: AP, AK, and AN; **(C)** organic matter (OM), Ca, and Mg. Values are the mean ± SD of three replicates. S_0_, initial soil; (S + EW)_0_, initial soil combined 10% (w/w) wormcast; (S + CD)_0_, initial soil combined 10% (w/w) cow dung compost; S, healthy control (healthy soil); S + F, disease control (healthy soil + *FOC*4); S + F + B, healthy soil + *FOC*4 + HN03 treatment; S + F + B + EW, healthy soil + *FOC*4 + HN03 + wormcast treatment; S + F + EW, healthy soil + *FOC*4 + wormcast treatment; S + F + B + CD, healthy soil + *FOC*4 + HN03 + cow dung compost treatment; S + F + CD, healthy soil + *FOC*4 + cow dung compost treatment. In the graph, capital letters indicate significant differences in original soil properties, and lowercase letters indicate significant differences in the potting soil in the different treatments. Bars sharing the same letter are not significantly different based on Tukey’s test at *P* < 0.05.

The mineral nutrient concentrations of the three environments, initial soil (S_0_), initial soil with wormcast (S + EW)_0_, and natural soil with cow dung compost (S + CD)_0_, were investigated before adding HN03. As shown in Figure 5, the TP, AP, AN, Ca, and Mg contents in (S + EW)_0_ were significantly higher than those in CD and S_0_, while the AK contents were highest in (S + CD)_0_. After the 3-month pot experiments, the contents of TP, AP, Ca, and Mg remained at high levels in the wormcast treatments (“S + F + B + EW” and “S + F + EW”), and when combined with HN03 (S + F + B + EW), the TK and AN contents reached the highest level. In contrast, the contents of AK and OM remained at high levels in the cow dung treatments (“S + F + B + CD” and “S + F + CD”), and the contents of TN and OM reached the highest levels when combined with HN03 (S + F + B + CD).

### Composition, Structure, and Functional Annotation of the Soil Microbial Community

The indices for community abundance, i.e., Chao 1 and ACE, and the indices for community diversity, i.e., Simpson and Shannon, were estimated (Table 1). The values of the Chao 1 and ACE indices for bacterial community abundance were significantly higher in the soil treated with HN03 than those in the “S + F” treatment, and the values were highest in the two treatments with wormcast. Additionally, the highest Shannon index and the lowest Simpson index were obtained in the “S + F + B + EW” treatment.

**TABLE 1:** The means of OTU, Chao 1, ACE, Simpson, and Shannon indices for soil bacteria (97% similarity) challenged with *FOC*4.

Treatment	OTUs	ACE	Chao1	Simpson	Shannon
S	1268 ± 10cd	1350.21 ± 34.85cd	1373 ± 32bc	0.0068 ± 0.0006a	5.94 ± 0.01cd
S + F	1159 ± 27d	1238.24 ± 31.2d	1246 ± 39c	0.0064 ± 0.0003a	5.81 ± 0.04d
S + F + B	1351 ± 11bc	1431.72 ± 36.62abc	1460 ± 47ab	0.0056 ± 0.0001ab	6.00 ± 0.02c
S + F + B + EW	1405 ± 31a	1487.95 ± 52.28ab	1505 ± 67a	0.0041 ± 0.0004c	6.30 ± 0.03a
S + F + EW	1388 ± 15ab	1487.88 ± 7.84a	1509 ± 12a	0.0054 ± 0.0011ab	6.14 ± 0.05b
S + F + B + CD	1320 ± 32bc	1425.97 ± 21.96abc	1461 ± 18ab	0.0059 ± 0.0005a	5.99 ± 0.09c
S + F + CD	1305 ± 45c	1405.38 ± 31.75bc	1437 ± 31abc	0.0062 ± 0.0005a	6.01 ± 0.02c

*Data are presented as the mean ± SD. Different letters in each column indicate significant differences on the basis of Tukey’s test (P < 0.05). S, healthy control (healthy soil); S + F, disease control (healthy soil + FOC4); S + F + B, healthy soil + FOC4 + HN03 treatment; S + F + B + EW, healthy soil + FOC4 + HN03 + wormcast treatment; S + F + EW, healthy soil + FOC4 + wormcast treatment; S + F + B + CD, healthy soil + FOC4 + HN03 + cow dung compost treatment; S + F + CD, healthy soil + FOC4 + cow dung compost treatment.*

**FIGURE 6:**
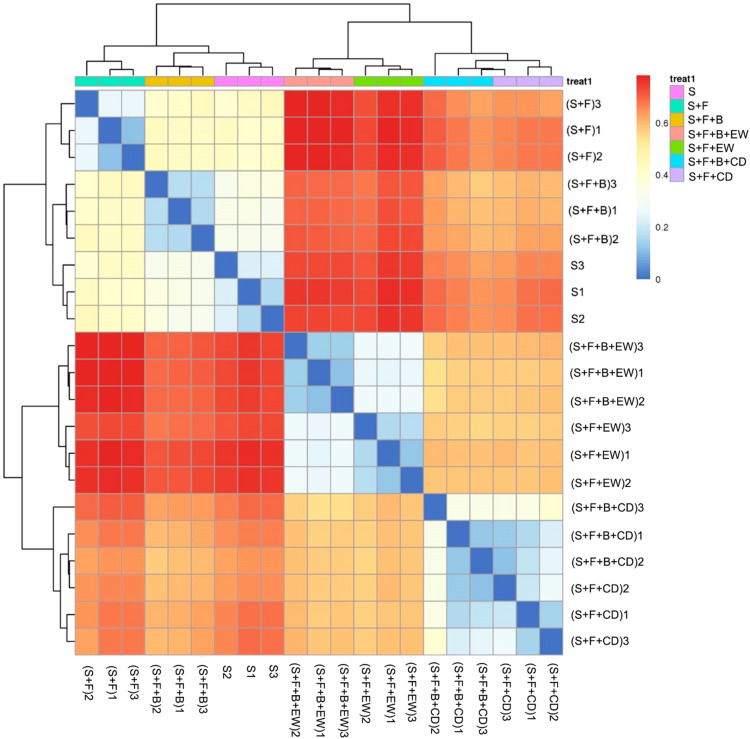
Heatmap of similarities between the bacterial communities based on Bray–Curtis distance indices in the different treatments. S, healthy control (healthy soil); S + F, disease control (healthy soil + *FOC*4); S + F + B, healthy soil + *FOC*4 + HN03 treatment; S + F + B + EW, healthy soil + *FOC*4 + HN03 + wormcast treatment; S + F + EW, healthy soil + *FOC*4 + wormcast treatment; S + F + B + CD, healthy soil + *FOC*4 + HN03 + cow dung compost treatment; S + F + CD, healthy soil + *FOC*4 + cow dung compost treatment. Every treatment has three replicates and was named treatment or (treatment) + numbers (1, 2, 3). The color gradient from red to blue indicates increasing similarity. Panels within the figures indicate the similarity among soil samples.

The heatmap analysis of the operational taxonomic units (OTUs) with hierarchical clustering based on the Bray–Curtis distance and Binary–Jaccard distance indicated that the community structural patterns differed significantly with respect to amendment types. In terms of bacteria (Figure 6), the treatments with cow dung compost or wormcast were clearly separated from the treatments without compost. For the groups in soil without compost, treatment “S + F” was clearly separated from “S and S + F + B.” Groups with compost were divided into two groups: one with wormcast and one with cow dung compost. With the addition of HN03, the wormcast groups were well separated, whereas the cattle manure groups were not as well separated. In addition, treatment “S + F + B + EW” had the lowest bacterial community similarity with that of treatment “S + F.” The grouping of fungal communities was similar to that of the bacterial communities (Supplementary Figure 1).

**FIGURE 7:**
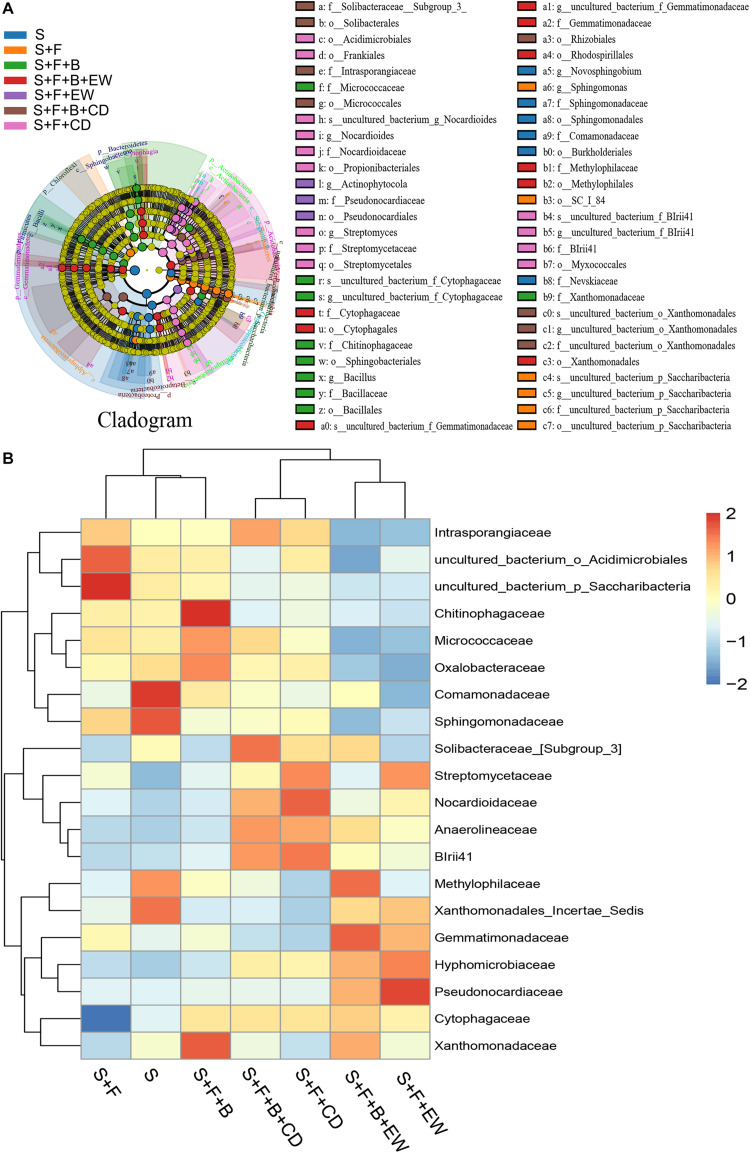
Taxonomic cladogram obtained from LEfSe analysis of 16S sequences (relative abundance *P* ≥ 0.5%) **(A)**; top-20 heatmap similarities between the bacterial communities based on Euclidean distance indices of the different treatments **(B)**. S, healthy control (healthy soil); S + F, disease control (healthy soil + *FOC*4); S + F + B, healthy soil + *FOC*4 + HN03 treatment; S + F + B + EW, healthy soil + *FOC*4 + HN03 + wormcast treatment; S + F + EW, healthy soil + *FOC*4 + wormcast treatment; S + F + B + CD, healthy soil + *FOC*4 + HN03 + cow dung compost treatment; S + F + CD, healthy soil + *FOC*4 + cow dung compost treatment.

According to the comparative analysis of the microbiome in the soil samples of all treatments, differences were observed in the community structure and abundance of specific family groups. The 16S rRNA gene data using the linear discriminant analysis (LDA) effect size (LEfSe) approach was used to further reveal the abundance of the top 20 bacterial families and identify the key phylotypes among the treatments. The results are shown in Figure 7. Compared with the healthy soil without *FOC4*, the abundance of Comamonadaceae, Solibacteraceae, Methylophilaceae, Xanthomonadaceae_Incerae_Sedis, Cytophagaceae, and Xanthomonadaceae was decreased significantly in the soil of the “S + F” treatment, and the abundance of Cytophagaceae was the lowest among all treatments. In the soil inoculated with *FOC4*, the bacterial families Comamonadaceae, Methylophilaceae, and Xanthomonadaceae were recovered in the treatments with HN03, and the highest abundances of Xanthomonadaceae, Chitinophagaceae, Micrococcaceae, and Oxalobacteraceae were found in the “S + F” treatment. The highest abundance of Methylophilaceae was detected when HN03 was combined with wormcast. Cytophagaceae was significantly enriched in all amended treatments, especially in the treatment “S + F + B + EW.” Additionally, the abundance of Solibacteraceae and Xanthomonadaceae_Incerae_Sedis was mainly recovered in the treatments amended with cow dung compost and wormcast, respectively. On the contrary, the abundance of Intrasporangiaceae, uncultured_bacterium_o_Acidmicrobiales, and uncultured_bacterium_o_Saccharibacteria was increased significantly in the soil of the “S + F” treatment compared with the healthy soil. The abundance of these bacterial families was significantly suppressed by HN03 or wormcast, especially HN03 combined with wormcast.

**FIGURE 8:**
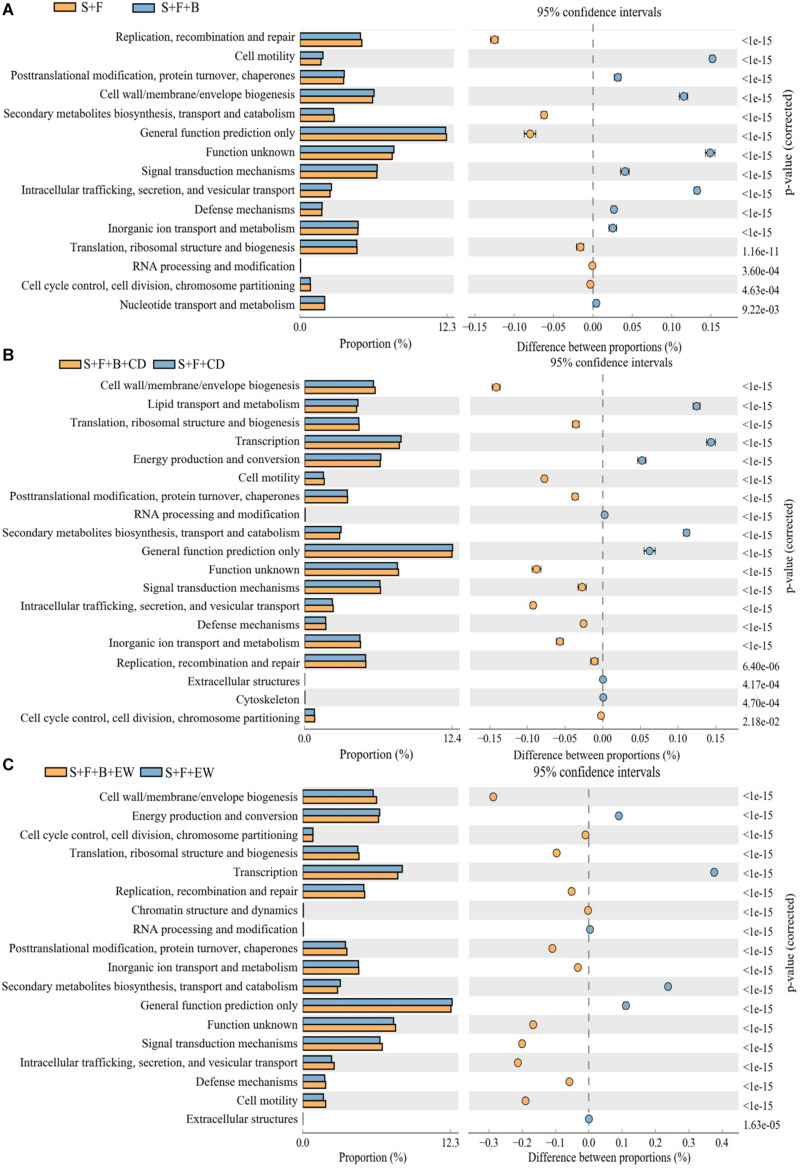
The cluster of orthologous groups (COG) categories differed significantly between treatments. “S + B” and “S + B + C” treatments **(A)**, “S + F + B + CD” and “S + F + CD” **(B)**, and “S + F + B + EW” and “S + F + EW” **(C)**. Values are the mean ± SD of three replicates. S, healthy control (healthy soil); S + F, disease control (healthy soil + *FOC*4); S + F + B, healthy soil + *FOC*4 + HN03 treatment; S + F + B + EW, healthy soil + *FOC*4 + HN03 + wormcast treatment; S + F + EW, healthy soil + *FOC*4 + wormcast treatment; S + F + B + CD, healthy soil + *FOC*4 + HN03 + cow dung compost treatment; S + F + CD, healthy soil + *FOC*4 + cow dung compost treatment. Bars sharing the same letter are not significantly different on the basis of Tukey’s test at *P* < 0.05.

Several COG categories were further exploration to differ significantly between these groups (Figure 8) indicating that at a broad scale these groups are metabolically and functionally distinct from each other. Examining individual COG categories in detail indicates that the “S + F + B + EW” contains relatively more genes assigned to categories defense mechanisms compared with the other treatments. The genes assigned to categories cell motility and signal transduction mechanisms in the treatment “S + F” were relatively less compared with the healthy soil, and were increased significantly in “S + F + B + EW” (Supplementary Figure 2). HN03 enhanced significantly different COGs within categories such as cell wall/membrane/envelope biogenesis; cell motility; posttranslational modification, protein turnover, and chaperones; intracellular trafficking, secretion, and vesicular transport; inorganic ion transport and metabolism; defense mechanisms; signal transduction mechanisms; and function unknown, while the highest abundance of cell wall/membrane/envelope biogenesis; cell motility, posttranslational modification, protein turnover, and chaperones; intracellular trafficking, secretion, and vesicular transport; inorganic ion transport; and metabolism.

### Associations of DI, Leaf and Soil Mineral Nutrition, and Microbes

**FIGURE 9:**
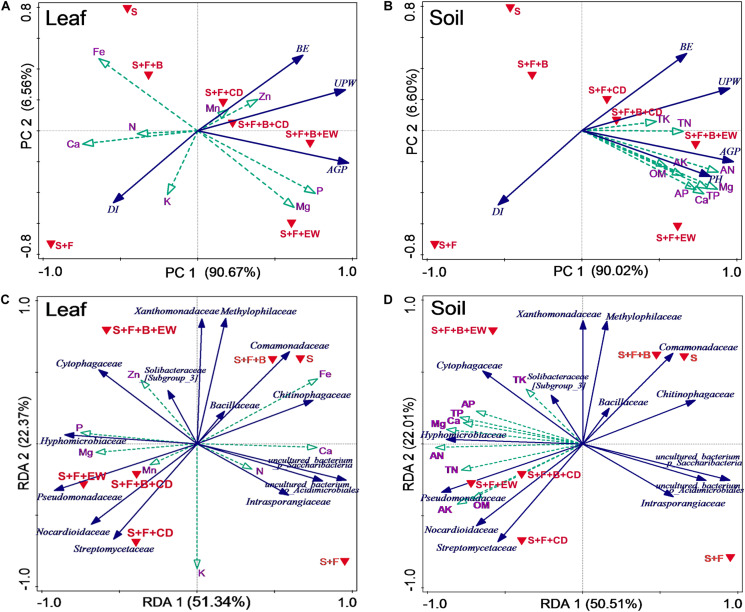
Associations of DI (disease incidence), leaf and soil mineral nutrients, and microbes. Principal component analysis (PCA) based on biocontrol and selected leaves **(A)** and soil **(B)** mineral nutrients in the different treatments. Variable included DI (disease incidence), BE (biocontrol efficacy), UPW (belowground weight), AGP (aboveground weight), and PH (pseudo-stem height). Redundancy analysis (RDA) based on the relative abundance of bacterial families and selected leaves **(C)** and soil **(D)** mineral nutrients in the different treatments. S, healthy control (healthy soil); S + F, disease control (healthy soil + *FOC*4); S + F + B, healthy soil + *FOC*4 + HN03 treatment; S + F + B + EW, healthy soil + *FOC*4 + HN03 + wormcast treatment; S + F + EW, healthy soil + *FOC*4 + wormcast treatment; S + F + B + CD, healthy soil + *FOC*4 + HN03 + cow dung compost treatment; S + F + CD, healthy soil + *FOC*4 + cow dung compost treatment.

According to the principal component analysis (PCA) (Figures 9A,B), the associations of DI, fresh weight, and BE with leaf mineral nutrition and soil mineral nutrition explained 97.23 and 96.62% of the variability, respectively, of all the data sets. The first component (PC1), which explained 90.67 and 90.02% of the total variation of DI associated with leaf and soil mineral nutrition, respectively, separated treatments “S,” “S + F,” and “S + F + B” from the treatments amended with cow dung compost or wormcast. The second component (PC2), which explained 6.56 and 6.6% of the total variation of DI associated with leaf and soil mineral nutrition, respectively, separated treatment “S + F” from treatments “S” and “S + F + B” and separated treatments “S + F + B + EW” and “S + F + EW” from treatments “S + F + B + CD” and “S + F + CD”.

With respect to the leaf, the highest P and Mg contents were in treatment “S + F + B + EW,” and the highest Mn and Zn contents were in treatment “S + F + B + CD.” The higher Zn and Mn contents in treatment “S + F + B” were negatively correlated with DI (Figure 9A). The nutrient concentrations of TP, TK, TN, AP, AK, AN, Ca, Mg, and OM in the soil were negatively correlated with DI, particularly the TK and TN contents (Figure 9B).

Redundancy analysis (RDA) showed that the first and second RDA components were able to explain 73.71 and 72.51% of the total bacterial variation in leaf and soil nutrients, respectively (Figures 9C,D). The first component (RDA1), explaining 51.34 and 50.51% of the total variation of bacterial families with leaf and soil nutrients, respectively, separated the treatments “S,” “S + F,” and “S + F + B” from treatments amended with cow dung compost or wormcast. The second component (RDA2), explaining 22.37 and 22.01% of the total variation of bacterial families with leaf and soil nutrients, respectively, separated treatment “S + F” from treatments “S” and “S + F + B,” and treatment “S + F + B + EW” from treatments “S + F + B + CD,” “S + F + CD,” and “S + F + EW.”

The similarity in Euclidean distance between “S” and “S + F + B” was high, whereas the lowest similarity was detected between “S + F” and “S + F + B + EW” (Figure 9), which was consistent with the similarity of the bacterial groupings (Figure 6). When the plant and associated soil were threatened by *FOC*4, the addition of HN03 reshaped the elements and soil community structure into a healthy environment. In particular, the DI values were lower when HN03 was combined with wormcast.

The dominant bacterial communities were associated with soil nutrients in the different environments. Spearman’s correlation analysis results based on the selected soil chemical properties and the top-20 bacterial families and Bacillaceae family abundance results revealed that among the candidate bacterial families, the abundance of Bacillaceae, of which the highest abundance occurred in treatment “S + F + B,” was not correlated with nutrient elements in the soil. However, the abundances of Intrasporangiaceae, uncultured_bacterium_o_Acidimicrobiales, and uncultured_bacterium_o_Sphingobacteriales were negatively correlated with TP and Mg in the soil, and the highest abundance of these families occurred in the treatment “S + F,” while the lowest occurred in the treatments of “S + F + EW” and “S + F + B + EW.” Moreover, the contents of TP and Mg were higher in the treatments containing wormcast than the other treatments (Figure 5). The highest abundance of Gemmatimonadaceae was found in the treatments with wormcast and was positively correlated with TK (Table 2), while the concentration of TK was highest in the treatment “S + F + B + EW” (Figure 5).

**TABLE 2:** Spearman’s coefficients of correlation (*r*) between 21 bacterial families (Bacillaceae and the top 20 families in abundance) and soil properties.

No.	Family	AK	AP	AN	TK	TP	TN	OM	Ca	Mg
1	Intrasporangiaceae	−0.02	−0.40	−0.47^b^	−0.78	−0.56^a^	−0.11	0.06	−0.52^b^	−0.50^b^
2	uncultured_bacterium_o_Acidimicrobiales	−0.60^a^	−0.69^a^	−0.77^a^	−0.41	−0.78^a^	−0.64^a^	−0.59^a^	−0.72^a^	−0.72^a^
3	uncultured_bacterium_o_Sphingobacteriales	−0.59^a^	−0.79^a^	−0.88^a^	−0.52^b^	−0.88^a^	−0.70^a^	−0.58^a^	−0.83^a^	−0.84^a^
4	Chitinophagaceae	−0.70^a^	−0.75^a^	−0.66^a^	−0.33	−0.66^a^	−0.61^a^	−0.77^a^	−0.68^a^	−0.66^a^
5	Micrococcaceae	−0.46^b^	−0.78^a^	−0.70^a^	−0.69^a^	−0.69^a^	−0.35	−0.45^b^	−0.69^a^	−0.67^a^
6	Oxalobacteraceae	−0.52^b^	−0.87^a^	−0.73^a^	−0.48^b^	−0.74^a^	−0.37	−0.62^a^	−0.79^a^	−0.76^a^
7	Comamonadaceae	−0.61^a^	−0.69^a^	−0.53^b^	−0.09	−0.61^a^	−0.43	−0.71^a^	−0.69^a^	−0.64^a^
8	Sphingomonadaceae	−0.55^b^	−0.69	−0.85	−0.45^b^	−0.86	−0.53^b^	−0.46^b^	−0.81	−0.80
9	Solibacteraceae_[Subgroup_3]	0.22	0.14	0.16	−0.08	0.04	0.35	0.25	−0.04	0.03
10	Streptomycetaceae	0.54^b^	0.41	0.31	−0.06	0.38	0.39	0.55^b^	0.48^b^	0.47^b^
11	Nocardioidaceae	0.91^a^	0.64^a^	0.66^a^	0.08	0.65^a^	0.84^a^	0.84^a^	0.68^a^	0.69^a^
12	Anaerolineaceae	0.82^a^	0.64^a^	0.81^a^	0.20	0.75^a^	0.86^a^	0.72^a^	0.71^a^	0.71^a^
13	BIrii41	0.86^a^	0.50^b^	0.69^a^	0.18	0.62^a^	0.93^a^	0.71^a^	0.57^a^	0.60^a^
14	Methylophilaceae	−0.55^b^	−0.12	−0.05	0.28	−0.11	−0.38	−0.50^b^	−0.17	−0.14
15	Xanthomonadales_Incertae_Sedis	−0.50^b^	0.19	−0.01	0.44^b^	0.07	−0.36	−0.25	0.10	0.07
16	Gemmatimonadaceae	−0.32	0.37	0.33	0.44^b^	0.39	−0.31	−0.24	0.36	0.39
17	Hyphomicrobiaceae	0.52^b^	0.84^a^	0.83^a^	0.35	0.90^a^	0.56^a^	0.60^a^	0.89^a^	0.93^a^
18	Pseudomonadaceae	0.79^a^	0.74^a^	0.65^a^	0.19	0.68^a^	0.69^a^	0.83^a^	0.73^a^	0.70^a^
19	Cytophagaceae	0.31	0.32	0.56^a^	0.23	0.55^b^	0.48^b^	0.21	0.44^b^	0.59^a^
20	Xanthomonadaceae	−0.44^b^	−0.14	0.12	0.16	0.13	−0.12	−0.46^b^	0.05	0.10
21	Bacillaceae	0.28	−0.13	0.18	−0.05	0.18	0.30	−0.02	0.12	0.22

*^a^Correlation is significant at P < 0.05. ^b^Correlation is significant at P < 0.01 A, available; T, total; OM, organic matter.*

## Discussion

### Characteristics of HN03 and the Effects of Its Application on the Suppression of *Fusarium* wilt

The HN03 strain isolated from the soil was molecularly identified as *B. velezensis*. According to the results of the biochemical tests, the strain could utilize a wide range of carbon sources and physiological and biochemical characteristics that helped it adapt to the environment. *B. velezensis* strains have good potential for biocontrol and can promote plant growth (Cai et al., 2017; Liu et al., 2017), and they are also effective against *F. oxysporum* (Moreno-Velandia et al., 2018). In this study, we demonstrated that HN03 has antagonistic activity against a wide spectrum of pathogenic fungi, with inhibition rates ranging from 44.12 to 77.62%. We also found that HN03 promoted the growth of the banana seedlings, especially the underground parts, even though the banana seedlings were infected with *FOC*4, which always infects banana from the root to the rhizome during the early infection stages (Ploetz, 2006). Moreover, when we studied the effects of organic fertilizer (wormcast or cattle manure), HN03 (10:1, w/w, spores > 10^8^ CFU/mL), or their combination on banana growth and the suppression of *Fusarium* wilt in pot trials, we found that HN03 and compost reduced the DI; the combination of HN03 and cow dung compost showed a lower DI; and the combination of HN03 and wormcast showed the lowest DI (Figure 3B). In this study, different composts differentially impacted the nutrients and community in the soil to induce soil suppression, which may have induced plant resistance. HN03 reestablished the community structure in the soil to induce soil suppression and induced plant resistance by enhancing the POD contents and expression of Zn. When combined with compost, HN03 altered the mechanisms by which the compost acts by changing the types of nutrients, enhancing the POD contents, and modulating the community structure, thereby further inducing soil suppression and plant resistance. The comprehensive mechanisms are discussed below.

### Mechanisms Related to the Resistance Activity of the Plant

To determine the differences in the mechanisms for the biocontrol of *Fusarium* wilt in banana plants by HN03 in different environments, we tested for POD in the leaves, as these compound is associated with disease resistance in plants (Su et al., 2016). POD participates in the construction, rigidification, and eventual lignification of cell walls to protect plant tissues from damage (Sun et al., 2012). In a previous study, a resistant cultivar of banana had an inherently higher capacity to stimulate POD production than a susceptible cultivar (Aguilar et al., 2000). Moreover, *B. velezensis* can trigger basal immunity in plants (Jiang et al., 2018) by increasing the expression of plant defense-related genes and the activities of some defense enzymes, such as catalase (CAT) and POD (Jiang et al., 2019). In this study, banana seedlings treated with HN03 had a higher POD content than those in the treatment without HN03, and the highest POD content was detected in the treatment with HN03 combined with wormcast. Thus, HN03 combined with wormcast could induce resistance to pathogen infection by increasing POD activity in banana, which is similar to the mechanism exhibited by disease-resistant cultivars when threatened by the pathogen. To summarize, a high POD content in the plants may be a key factor in suppressing banana vascular wilt disease when HN03 or “HN03 with wormcast” is applied to the soil. Only a suitable carrier can trigger this mechanism, as the POD content is low in the cow dung compost.

### Mechanisms Related to Nutrient Element Modulation

In our study, a positive correlation was found between the contents of Zn, Mn, Mg, and P in banana and the suppression of wilt disease severity, which is consistent with the results of Hassan and Abo-Elyousr (2013) and Siddiqui et al. (2015). Some nutrient elements in banana and its associated soil can reduce the severity of plant disease by increasing disease tolerance and resistance against plant pathogens (Dordas, 2008; Siddiqui et al., 2015). Mn, which was significantly higher in the treatments with HN03 than in their controls, can be a highly effective micronutrient in inducing plant resistance against diseases by affecting cell wall composition, lignin biosynthesis, phenol biosynthesis, photosynthesis, and several other functions (Hassan and Abo-Elyousr, 2013). It also suppresses the penetration of pathogens into plant tissue and accumulates in the form of Mn^4+^ at the sites where pathogens attack (Dordas, 2008). High Zn levels in leaf tissues are associated with the strong suppression of wilt disease because of the direct toxic effects of Zn on pathogens (Dordas, 2008). The highest contents of Mn and Zn were found in the “S + F + B + CD” treatment. Mg and P, which were the highest in the “S + F + B + EW” treatment, can affect the suppression of plant diseases both directly by affecting pathogen growth and indirectly by affecting plant defenses and stomatal functions (Walters and Bingham, 2007; Huber and Jones, 2012). Therefore, it can be concluded that HN03 can improve the organic amendment strategy in suppressing *Fusarium* wilt of banana plants by modulating Mn. Wormcast is helpful for the accumulation of Mg, P, and Zn in the banana leaves to enhance plant suppression, while cow dung compost can induce plant suppression to *FOC4* by modulating Mn and Zn. HN03 combined with wormcast or cow dung compost can significantly promote Mg/P or Mn/Zn assimilation, respectively, in the leaves.

The contents of TP, TK, and AN in the soil are correlated with increased production, decreased pathogen infection, and reduced disease severity in susceptible crops (Brennan, 1995; Walters and Bingham, 2007; Shen et al., 2015). High N and K contents in the soil decreased the severity of *F. oxysporum* infection (Dordas, 2008). Similarly, in our study, a positive correlation was found between the AN and TK contents in the soil and the suppression of wilt disease. We also found that the contents of TP and TK were higher in the treatments of “S + F + B + EW” and “S + F + EW” than in the other treatments, and AN was higher in the treatments with wormcast or cow dung compost. Additionally, the contents of TK or AN increased significantly when HN03 was combined with wormcast or cow dung compost, respectively. By contrast, the contents of TN and OM, which are negatively correlated with DI and promote the uptake of Mn and Zn by higher plants (Siddiqui et al., 2015), were higher in the treatments of “S + F + B + CD” and “S + F + CD” than in the other treatments, and were highest in “S + F + B + CD.” Notably, the contents of OM and TN in treatment “S + F + B + CD” were even higher than in both the unplanted soil treated with cow dung compost (S + CD)_0_ and the compost control (S + F + CD). These results indicated that HN03 could decompose some substances, such as microbial or plant residue, and thereby increase the nutrient content in the soil.

We concluded that HN03 regulated soil nutrients according to the soil environment and, as a result, suppressed the pathogen in the soil and adjusted the uptake of plant nutrients, eventually inducing plant resistance against the pathogen. HN03 increased the contents of Mn and Zn in the plants when used alone. In addition, the contents of Mn and Zn in the plants were maximized when HN03 was combined with cow dung compost. The high content of OM in the soil of treatment “S + F + B + CD” could explain this result, which by facilitating Mn and Zn absorption, ultimately led to *FOC4* resistance. The content of P and Mg in the plants increased in the treatments with wormcast, which could be explained by the higher contents of TP, AP, and Mg in the wormcast. When treated with HN03 combined with wormcast, the soil had higher AN and TK contents; therefore, HN03 may suppress *FOC*4 infection by increasing the N and K contents.

### Mechanisms Related to Soil Microbiome Modulation

Reshaping of the soil microbiome is the main mechanism by which soil suppression against *Fusarium* wilt disease is induced and has been widely discussed in bio-control systems (Wang et al., 2016; Xiong et al., 2017). According to the results of our study, in the treatment with HN03, the abundance (ACE and Chao 1 indices) and the diversity (Simpson and Shannon indices) of bacterial communities in the soil increased significantly compared with those in the “S + F” treatment. When HN03 was combined with wormcast, the abundance and the diversity of the bacterial communities in the soil peaked. Moreover, the community structure in the soil samples inoculated with *FOC*4 tended to be similar to that in healthy soil after being treated with HN03 for 90 days. The effect of the wormcast treatment on community structure was greater than that of the cow manure treatment. In addition, HN03 and wormcast application increased the differences in bacterial community structure in the soil samples inoculated with *FOC*4, compared with little influence on the soil samples with cow manure. Therefore, the wormcast was better than the cow manure in reshaping the community structure when the soil was infected with *FOC*4, and HN03 enhanced this effect.

The identification of key microorganisms is proposed as a first step in rebuilding the microbiome of tissue-culture banana plants prior to planting to improve defense responses against *FOC* (Dita et al., 2018). In our study, HN03 influenced the soil community structure and mobilized different dominant strains against pathogens in different soil environments. As reported in the literature, the bacteria families Solibacteraceae and Cytophagaceae are highly abundant in a *F. oxysporum*-resistant cultivar, and Cytophagaceae was identified as a *F. oxysporum-*suppressive bacterial taxon by Mendes et al. (2018). Comamonadaceae are a promising group of biocontrol microbes that are likely contribute to the recovery observed in plant growth (Durán et al., 2018) and were thus observed at higher abundance in all HN03 treatments. Members of Xanthomonadaceae, associated with the suppression of disease in soil (Grunert et al., 2016), and Chitinophagaceae, associated with plant growth promotion (Madhaiyan et al., 2015), were most abundant following treatment by HN03. The results shown in Figure 7B indicated that (1) strain HN03, isolated in our laboratory, can facilitate the growth of Comamonadaceae, Methylophilaceae, Cytophagaceae, and Xanthomonadaceae in the soil even under *FOC4* infection, and the function of Methylophilaceae could be enhanced by wormcast; (2) cow dung compost and wormcast can modulate the abundance of Solibacteraceae and Xanthomonadaceae_Incertae Sedis, respectively, and the abundance of Solibacteraceae was higher when cow dung compost was accompanied by HN03; (3) Cytophagaceae abundance can be increased by cow dung compost, wormcast, as well as HN03, and the abundance was highest in the treatment amended with HN03 and wormcast. In addition, some other bacterium families that produce erythromycin or are associated with disease suppression in the natural soil were enhanced by cow dung compost or wormcast, such as Nocardioidaceae (Harrell and Miller, 2016) and Pseudomonadaceae (Grunert et al., 2016). Furthermore, the abundance of Gemmatimonadaceae, which forms calcium carbonate via biomineralization and increases soil pH to improve soil quality (Wang et al., 2014), was found to be regulated by the TK of treatment “S + F + B + EW.” Obviously, the abundances of bacteria families associated with the suppression of *Fusarium* wilt of banana could be adjusted according to the different soil nutritional environments.

The mechanisms by which HN03 combined with different environments provided protection were further explained by the functional annotation results. Cell motility measures the capacity of the cells to translocate onto a solid substratum (Jouanneau and Thiery, 2002), and this trait is associated with increased colonization of biocontrol bacteria in the plant roots (Liddell and Parke, 1989). The cell motility in the treatments of “S + F + B” and “S + F + B + EW” was high probably because the biocontrol microbial community colonized the diseased plant roots and soil, which improved the suppression of the disease by the biocontrol microbial community. Signal transduction mechanisms, which can recognize specific signals and convert information into specific transcriptional or behavioral responses and thus help the microbial community to survive and prosper in a wide variety of environments (Fabret et al., 1999), were higher in the treatments with HN03 and were highest in the treatment with HN03 combined with wormcast. Relatively more genes assigned to categories inorganic ion transport and metabolism was found in all HN03 treatments, which may demonstrated the function of HN03 in regulating soil and plant nutrients. Compared with compost treatment only, HN03 or its combination with compost was associated with more functional traits, which are processes related to microorganism vital activities such as evolution (Brown et al., 2001). In our study, relatively more genes affiliated with categories defense mechanisms was contained in “S + F + B + EW” than in the other treatments, and consequently, the combination of HN03 and wormcast could regulate a dynamic community with high adaptation and colonization and therefore reduce DI through an increase in defensive mechanisms.

## Conclusion

**FIGURE 10:**
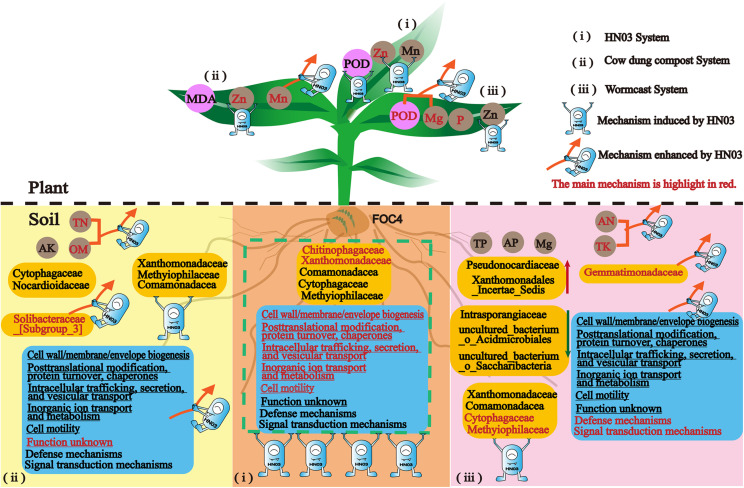
Conceptual model summarizing the mechanisms by which HN03 and its organic carriers defend against *Fusarium* wilt in banana.

In this work, we unraveled the mechanisms used by a new isolated biocontrol bacterium *B*. *velezensis* HN03 to fight banana *Fusarium* wilt in three types of soil environments: soil with the pathogen only and soil with the pathogen and cow dung compost or wormcast. The strain HN03 could reshape the soil community structure and microbiota motility, regulate soil nutrients to suppress disease, and induce plant resistance to *Fusarium* wilt, such as defense enzymes and nutrient elements. Furthermore, HN03 could alter the strategy by which compost controls soil-borne disease by enhancing the advantages of the composts and stimulating new mechanisms in the plants and soil (Figure 10).

A combination of biocontrol bacterium and carrier should thus be considered for enhancing plant defense and soil suppression when controlling soil-borne diseases. The right combination can stimulate plant defense responses by mobilizing specific plant enzymes and nutrient factors to disease and enhance soil suppression by regulating the microbial community and nutrient environment in the soil.

## Data Availability Statement

The datasets presented in this study can be found in online repositories. The names of the repository/repositories and accession number(s) can be found below: https://www.ncbi.nlm.nih.gov/genbank/, MF155192.

## Author Contributions

CW and XW contributed to the conception and design of the study. XW performed the experiments, organized and analyzed the data, and performed the writing the original draft preparation. CW contributed with conceptualization, writing – review and editing, and funding acquisition. YS and YL contributed to organizing and analyzing the data. QL contributed to the supervision. All authors read and approved the final manuscript.

## Conflict of Interest

The authors declare that the research was conducted in the absence of any commercial or financial relationships that could be construed as a potential conflict of interest.

## Footnotes

1 https://www.ncbi.nlm.nih.gov

2 https://www.ezbiocloud.net

3 www.biocloud.net

4 http://huttenhower.sph.harvard.edu/lefse/
