# Supplementary figures and images for: The Soil Nutrient Environment Determines the Strategy by Which Bacillus velezensis HN03 Suppresses Fusarium wilt in Banana Plants
Source: Front Plant Sci. 2020 Nov 16;11:599904. doi: 10.3389/fpls.2020.599904 (PMC7701294; doi:10.3389/fpls.2020.599904)

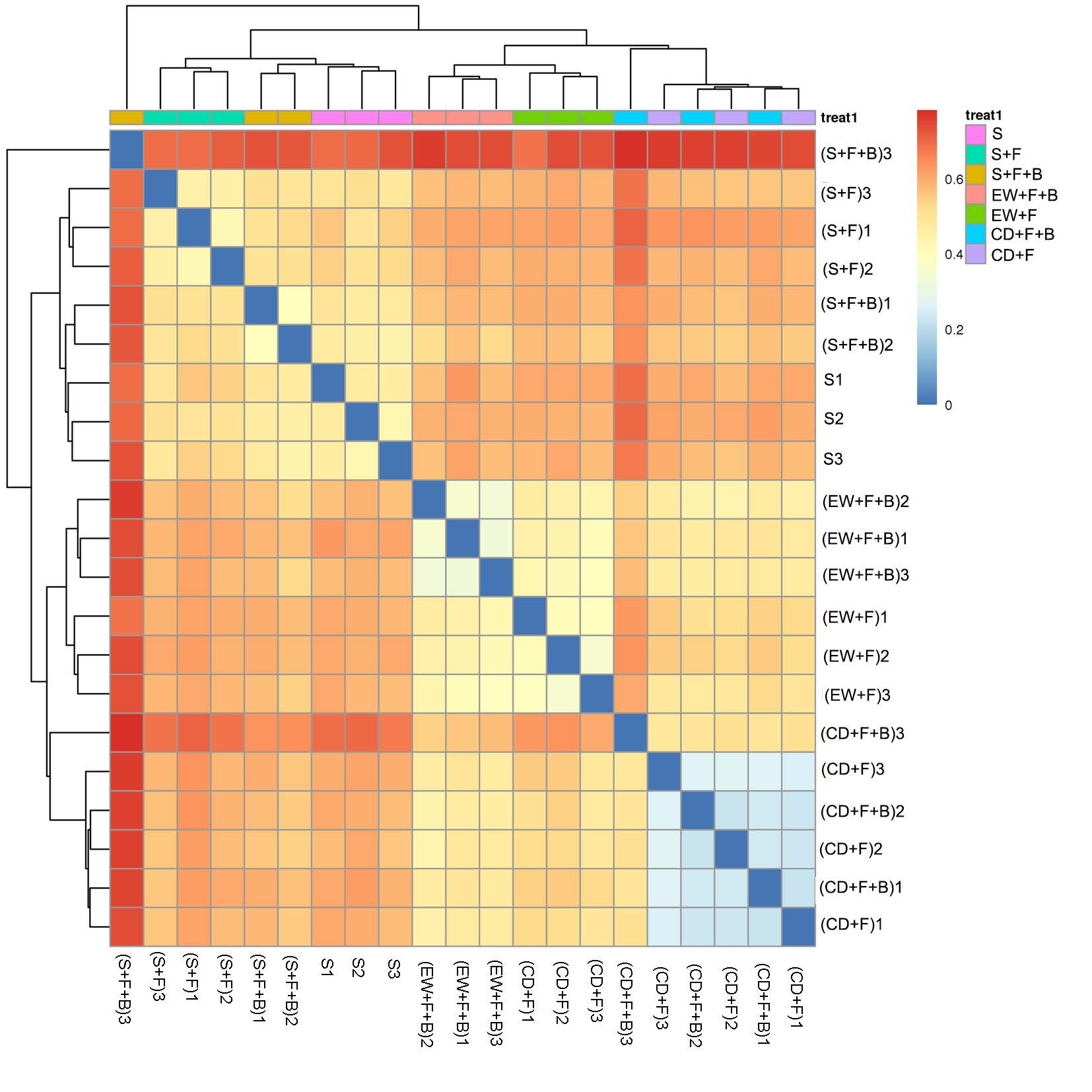

Supplement: Supplementary file 1 [file Image_1.TIF]

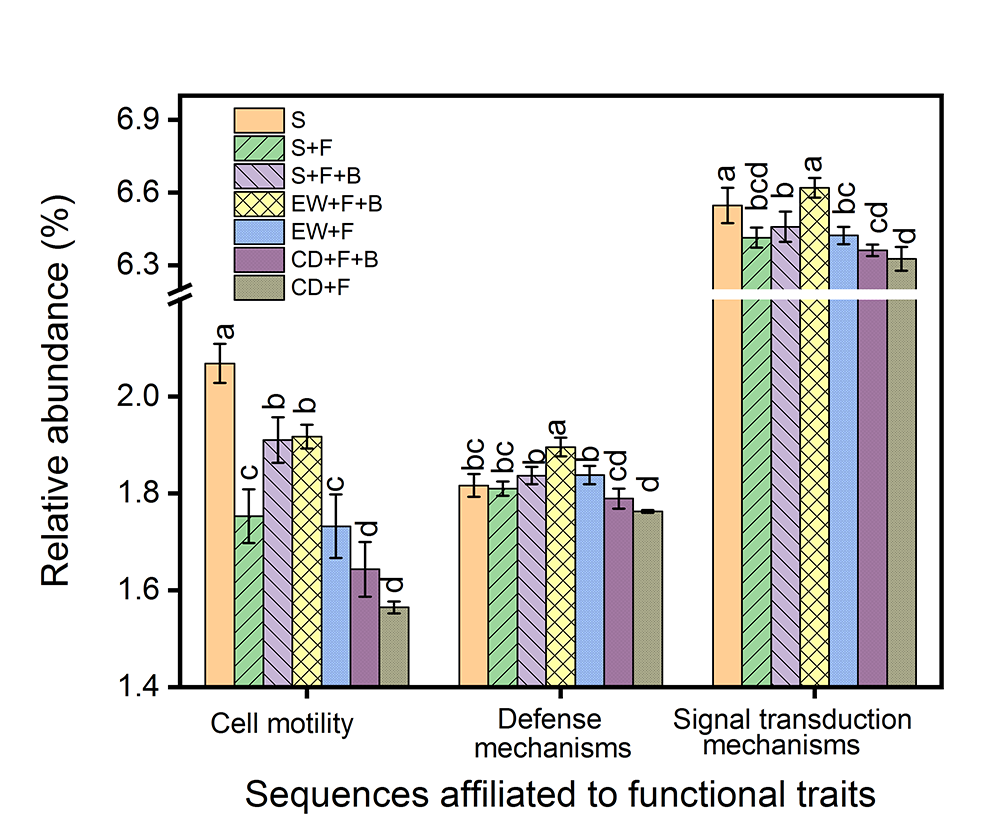

Supplement: Supplementary file 2 [file Image_2.TIF]
