# Supplementary material for: The Soil Nutrient Environment Determines the Strategy by Which Bacillus velezensis HN03 Suppresses Fusarium wilt in Banana Plants
Source: Front Plant Sci. 2020 Nov 16;11:599904. doi: 10.3389/fpls.2020.599904 (PMC7701294; doi:10.3389/fpls.2020.599904)
Supplement: Supplementary file 3 [file Table_1.DOCX]

# Supplementary Table 1. Carbon source utilization and chemical sensitivity assays for strain HN03 using the GenIII Microplate.

| Characteristic | Result | Characteristic | Result | Characteristic | Result | Characteristic | Result |
| --- | --- | --- | --- | --- | --- | --- | --- |
| Dextrin | + | Fusidic acid | - | Guanidine HCl | w | α-Keto-glutaric acid | w |
| D-maltose | + | D-serine | - | Niaproof 4 | - | D-malic acid | - |
| D-cellobiose | + | D-glucose-6-PO4 | w | D-galacturonic acid | + | L-malic acid | + |
| Stachyose | w | D-Fructose-6-PO4 | w | L-galactonic acid Lactone | + | Bromo-succinic acid | + |
| α-D-lactose | - | D-aspartic acid | + | D-Gluconic Acid | + | Nalidixic acid | - |
| β-Formyl-D-glucoside | + | Troleandomycin | - | D-Glucuronic Acid | w | Lithium Chloride | + |
| D-salicin | + | Rifamycin SV | - | Glucuronamide | - | Potassium tellurite | + |
| N-Acetyl-D-glucosamine | + | Minocycline | - | Mucic Acid | w | γ-Amino-Butryric acid | w |
| N-acetyl-β-D Mannosamine | + | Glycyl-L-prolin | w | Quinic Acid | w | α-Hydroxy-butyric acid | - |
| N-acetyl-D-galactosamine | - | L-alanine | + | D-Saccharic Acid | w | β-Hydroxy-D,Lbutyric acid | - |
| N-acetyl Neuraminic Acid | - | L-arginine | + | Vancomycin | - | α-Keto-Butyric acid | - |
| α-D-Glucose | + | L-aspartic acid | + | Tetrazolium Violet | - | Acetoacetic acid | w |
| 3-Methyl glucose | - | L-glutamic acid | + | Tetrazolium Blue | - | Acetic acid | + |
| D-fucose | - | L-histidine | + | p-Hydroxy-Phenylacetic Acid | - | Formic acid | + |
| L-fucose | - | L-pyroglutamic acid | + | Methyl pyruvate | + | Aztreonam | + |
| inosin | w | L-serine | + | D-lactic acid methyl ester | - | Sodium Butyrate | - |
| 1% Sodium lactate | + | Lincomycin | - | L-lactic acid | + | Sodium Bromate | - |

+, Positive; -, Negative; W, Weak growth.
