# Supplementary material for: The Soil Nutrient Environment Determines the Strategy by Which Bacillus velezensis HN03 Suppresses Fusarium wilt in Banana Plants
Source: Front Plant Sci. 2020 Nov 16;11:599904. doi: 10.3389/fpls.2020.599904 (PMC7701294; doi:10.3389/fpls.2020.599904)
Supplement: Supplementary file 4 [file Table_2.DOCX]

**Supplementary Table 2.** Phenotypic characteristics of HN03 and 5 type strains of closely related species of the genus *Bacillus*

| Characteristic | 1 | 2 | 3 | 4 | 5 | 6 |
| --- | --- | --- | --- | --- | --- | --- |
| Gram-reaction | + | + | + | + | + | + |
| Anaerobic | - | - | - | - | - | + |
| Aerobic | + | + | + | + | + | + |
| Temperature for growth (℃): |  |  |  |  |  |  |
| Maximum | 50℃ | 50℃ | 50℃ | 45℃ | 45℃ | 55℃ |
| Minimum | 15℃ | 15℃ | 15℃ | 15℃ | 20℃ | 4℃ |
| pH values: |  |  |  |  |  |  |
| Minimum | 4.5 | ND | ND | 5 | 2 | 4.5 |
| Maximum | 10 | ND | ND | 10 | 10 | 9 |
| Voges–Proskauer Test | + | + | + | + | ND | + |
| Oxidase activity | + | + | + | + | + | - |
| Starch | + | + | + | + | ND | + |
| Gelatin | W | + | + | + | + | + |
| Twain-40 | - | + | + | ND | ND | ND |
| Soluble cellulose | + | - | + | ND | - | ND |
| Pectin | + | - | - | ND | ND | ND |
| Nitrate reduction | + | + | + | ND | + | + |
| Citrate utilization | + | + | + | - | ND | ND |
| Growth in the presence of NaCl (%): |  |  |  |  |  |  |
| 0.07 | + | + | + | + | - | + |
| 0.1 | + | + | + | + | - | + |
| Maximum | 10% | 10% | 10% | 12% | 4% | 14% |

Strains: 1, HN03; 2, *Bacillus amyloliquefaciens* DSM 7; 3, *Bacillus velezensis* FZB42T; 4, *Bacillus velezensis* CR-502; 5, *Bacillus methylotrophicus* CBMB205; 6, *Bacillus siamensis* KCTC13613=*Bacillus siamensis* strain PD-A10T =*Bacillus vanillea* XY18. Data were obtained in this study unless otherwise indicated. +, Positive; -, Negative; W, Weak growth; ND, Not determined. The used data of strain 2, 3, 4, 5, 6 were from the previous studies by Ruiz-Garcia et al. ([1](#_ENREF_1)), Madhaiyan et al. ([2](#_ENREF_2)), Sumpavapol et al. ([3](#_ENREF_3)), Borriss et al. ([4](#_ENREF_4)) respectively.
