# Supplementary material for: The Soil Nutrient Environment Determines the Strategy by Which Bacillus velezensis HN03 Suppresses Fusarium wilt in Banana Plants
Source: Front Plant Sci. 2020 Nov 16;11:599904. doi: 10.3389/fpls.2020.599904 (PMC7701294; doi:10.3389/fpls.2020.599904)
Supplement: Supplementary file 5 [file Table_3.DOCX]

**Supplementary Table 3.** HN03 inhibition of the mycelial growth of 12 pathogenic fungi on potato dextrose agar. Values are the mean ± standard deviation for 3 replicates.

| Plant pathogenic fungi | | Diameter of fungal colony (cm) | | Inhibition rate (%) |
| --- | --- | --- | --- | --- |
|  |  | Treatment | HN03 |  |
| 1 | *Fusarium oxysporum* f. sp. cubense tropical race 4 (*FOC*4) | 8.50 | 2.84±0.12 | 68.47±0.01 |
| 2 | *FOC*4（Vietnam） | 8.49 | 2.65±0.04 | 68.79±0 |
| 3 | *Fusarium oxysporum* f.sp*. cubense* 1 | 8.47 | 3.13±0.13 | 64.58±0.02 |
| 4 | *Fusarium solani* of Noni | 8.50 | 4.57±0.14 | 44.12±0.02 |
| 5 | *Fusarium solani* of Annona squamosal | 8.49 | 3.16±0.15 | 62.01±0.02 |
| 6 | *Fusarium oxysporum* f.sp*.radicis lycopersic* | 8.49 | 2.75±0.05 | 67.31±0.01 |
| 7 | *Fusarium oxysporum* f.sp.*melonis* | 8.50 | 2.55±0.02 | 70±0 |
| 8 | *Fusarium oxysporum* f.sp.*niveum* | 8.50 | 3.00±0.25 | 68.53±0.03 |
| 9 | *Phytophthora nicotianae* | 8.49 | 1.94±0.03 | 77.62±0 |
| 10 | *Colletotrichum gloeosporioides* | 8.50 | 1.89±0.08 | 76.47±0.01 |
| 11 | *Fusarium solani* of Medicago | 8.50 | 3.42±0.17 | 58.24±0.02 |
| 12 | *Fusarium solani* of Annona squamosal-1 | 8.50 | 3.12±0.12 | 62.94±0.01 |
